# The Effect of the Synthesis Method on Physicochemical Properties of Selective Granular Polymer Sorbents

**DOI:** 10.3390/polym14020353

**Published:** 2022-01-17

**Authors:** Alexandra Osipenko, Irina Garkushina

**Affiliations:** Institute of Macromolecular Compounds of the Russian Academy of Sciences, Bolshoy pr. 31, 199004 Saint Petersburg, Russia; osipeno4kalexa@mail.ru

**Keywords:** polymer sorbents, surface imprinting, Pickering emulsion polymerization, physicochemical properties, swelling kinetics, adsorption isotherms, Giles classification, isotherm models, surface affinity, sorption surface area

## Abstract

Investigation of the effect of the polymer synthesis method on physicochemical properties of sorbents is one of the topical problems in the chemistry of macromolecular compounds that has high scientific and practical interest. Determination of the optimal synthesis method will make it possible to create sorbents with physicochemical properties that led to the realization of effective sorption. In this work, we investigated the effect of synthesis methods (Pickering emulsion polymerization and precipitation polymerization in solution) of granular polymers based on 2-hydroxyethyl methacrylate and ethylene glycol dimethacrylate on physicochemical and sorption properties. The synthesis by Pickering emulsion polymerization led to improvement of the n-propyl alcohol diffusion into the polymer network and to the formation of more homogeneous and structurally stable polymer networks. Creating selective polymer networks by Pickering emulsion polymerization compared to precipitation polymerization in solution led to an increase in porosity, creation of more segregated surface of granules, improvement of binding sites availability at the temperature of 37 °C, and formation of the homogeneous sorption surface with high affinity to target molecules at 25 °C and 37 °C. Selective polymers synthesized by both polymerization methods had the largest values of available sorption surfaces areas for target molecules at 37 °C.

## 1. Introduction

Sorption materials are widely used in agriculture, industrial production, water treatment, and medicine to extract various impurities, pollutants, toxins, and metabolites [1,2,3], i.e., their use is closely related to environmental protection and public health. Sorbents are a large class of medical materials capable of removing toxic substances and metabolites from an organism. The development of effective sorption methods involves creating selective polymer sorbents.

One of the methods to create highly selective polymer sorbents is molecular imprinting [4,5]. Molecular imprinting consists of forming a polymer network in the presence of template molecules. For example, in case of non-covalent imprinting, template molecules are fixed in the polymer network due to non-covalent interactions: hydrogen bonds, electrostatic, hydrophobic, π-π, and van der Waals forces with functional monomers [6]. As a result, the crosslinked polymer after template extraction contains cavities complementary to its molecule and capable of selective re-binding [6,7]. Most studies on non-covalent imprinting for the synthesis of molecularly imprinted polymers (MIPs) use the method of radical polymerization with functional and crosslinking monomers containing acrylic and vinyl groups [8,9,10,11,12]. This is due to their variety and availability. The attractiveness of MIPs for practical application is due to such properties as ease of preparation, affinity and selectivity comparable to natural receptors, and low cost. Due to these properties, MIPs are increasingly being investigated for further use in various fields, including sensors, preparative sorption, water purification, medicine, etc. [13,14,15,16,17,18,19].

Selectivity of MIPs is due to the presence of complementary sorption centers in the polymer network that mimic natural receptors and, therefore, have a strong affinity for the target component. However, during the synthesis of MIPs, difficulties may arise, associated, for example, with a deep location or inaccessible imprint sites, which prevent the subsequent removal of the template and also contributes to formation of “defective” binding sites that reduces the efficiency of imprinting [20]. The solution to these problems is to create MIPs, in which the main part of the binding sites will be on the surface of the polymer sorbent [21,22]. 

The surface molecular imprinting method creates polymers with a high affinity of sorption surfaces [23]. The synthesis of polymers by surface imprinting not only increases the sorption capacity, but also avoids problems with the diffusion barrier and delayed mass transfer of target molecules [24,25,26,27]. As studies have shown [27,28,29], surface molecularly imprinted materials synthesized in the form of granules are more efficient for the recognition of target molecules.

A widely used method for producing granular polymers is emulsion polymerization [30]. However, the main disadvantage of this synthesis method is associated with the impossibility of purifying the final product from the surfactant. In addition, many surfactants are toxic, and, therefore, polymer products obtained with their use will be unsuitable for medical and biological applications.

The use of solid particles, instead of surfactants, to stabilize polymer-monomer droplets will lead to decrease in the toxicity of the obtained polymers and, consequently, to an increase in areas of their further use. Emulsion polymerization using solid particles as stabilizers for polymer-monomer droplets is called Pickering emulsion polymerization. In addition, compared to traditional emulsions stabilized by surfactants, Pickering emulsions not only retain basic properties of emulsions, but also demonstrate additional advantages, for example, higher coalescence resistance and low toxicity [31,32,33,34,35]. 

Pickering emulsion polymerization is a promising area for use in a wide range of areas: cosmetics, pharmaceuticals, green chemistry, food industry, medicine, etc. [36]. In medicine, one of the widespread diseases requiring the use of selective sorbents for efferent methods of treatment is familial hypercholesterolemia [37]. Accumulation of cholesterol in the body and it level in human blood over 6.21 mmol/L leads to development of atherosclerosis, which is one of the main causes of death in developed countries. A significant decrease in blood cholesterol levels is possible by means of pharmacotherapy with lipid-lowering drugs and diet. However, there is a special category of patients with homo- and heterozygous forms of familial hypercholesterolemia, in whom extremely high blood cholesterol levels persist even after drug therapy. To solve these problems, efferent methods of treatment (hemo- and plasmasorption) are now widely used [37,38]. These methods are based on column sorption processes carried out using hemosorbents as chromatographic carriers. Therefore, the creation of highly selective hemosorbents for the treatment of hypercholesterolemia is urgent.

In most work on the creation of sorbents, attention has been focused precisely on the method of synthesis [39,40]. However, little attention has been paid to the study of their physicochemical properties, such as swelling, density (bulk and true) in the solvated state, structural stability, homogeneity, porosity, etc. Various methods of synthesis lead to the creation of polymers with different physicochemical characteristics that, in turn, are reflected in their sorption properties. 

Consequently, the aim of this study was to identify a method for the synthesis of polymers that makes it possible to obtain selective polymer sorbents for cholesterol with optimal physicochemical properties. These sorbents can be used in the future for highly selective extraction of total cholesterol from blood plasma in vivo in the treatment of hypercholesterolemia.

## 2. Materials and Methods

### 2.1. Materials

Reagents and substances: selenous acid (H_2_SeO_3_) 98% («Acros Organics», Fair Lawn, NJ, USA), poly(vinylpyrrolidone) (PVP) (M_w_ 55 × 10^3^) («Ferak», Berlin, Germany), cholesterol 99%, cholic acid 97% («Acros Organics», New Jersey, USA), n-propyl alcohol (reagent grade) («Vekton», St. Petersburg, Russia), n-butyl alcohol (reagent grade) («Vekton», St. Petersburg, Russia), and Cholesterol-Vital test solution («Vital Development Corporation» JSC, St. Petersburg, Russia). 

Monomers: 2-hydroxyethyl methacrylate (2-HEMA) 98% and ethylene glycol dimethacrylate (EGDMA) 98% were used without pretreatment. 

Initiators: ascorbic acid (C_6_H_8_O_6_) («Vekton», St. Petersburg, Russia) and ammonium persulfate («Vekton», St. Petersburg, Russia) were used without pretreatment.

### 2.2. The Synthesis of Polymer Sorbents

#### 2.2.1. The Synthesis by Pickering Emulsion Polymerization

Polymer sorbents synthesized using nanoparticles were obtained by the free radical emulsion polymerization in Pickering emulsions [13], hereafter referred to as “Method 1” (MIP-1). A specific feature of this method of polymer synthesis was the use of Se^0^ (NP-Se) nanoparticles stabilized with poly(vinylpyrrolidone) as stabilizers for polymer-monomer droplets.

Synthesis of Se^0^ nanoparticles stabilized by poly(vinylpyrrolidone) (NP-Se/PVP nanocomplexes) was carried out by reduction of selenous acid (H_2_SeO_3_) with ascorbic acid (C_6_H_8_O_6_) in the aqueous solution of PVP to obtain red amorphous Se^0^ [41]:

(1) $$H_{2}{\mathrm{SeO}}_{3}+2C_{6}H_{8}O_{6}\to {\mathrm{Se}}^{0}+3H_{2}O+2C_{6}H_{6}O_{6}.$$

Nine milliliters of distilled water, 3 mL of 0.1635 wt.% aqueous solution of H_2_SeO_3_, and 3 mL of 0.447 wt.% aqueous solution of C_6_H_8_O_6_ was consistently introduced into a heat-resistant beaker with 25 mL of 0.2 wt.% PVP aqueous solution (M_w_ = 55 × 10^3^). The colloidal solution was kept at room temperature for 20 min, and a change in the color of the solution from transparent colorless to orange, corresponding to the color of colloidal selenium, was observed. The formation of selenium nanoparticles [42] was monitored spectrophotometrically at a wavelength of λ = 250−260 nm.

The synthesis of polymers was carried out similarly to [13]: 2-HEMA and EGDMA were sequentially introduced into a colloidal system containing NP-Se/PVP nanocomplexes with constant stirring. In contrast to [13], the ratio of HEMA and EGDMA monomers was 86 mol.% and 14 mol.%, respectively. A decrease in the amount of EGDMA to 14 mol.% should contribute to a decrease in the number of nonspecific binding sites for the cholesterol molecule. The total concentration of monomers in the solution was 30 wt.%. The synthesis of polymers was carried out in a three-necked round-bottom flask equipped with a mechanical stirrer.

The polymerization reaction was initiated by the system of ammonium persulfate-ascorbic acid in the weight ratio of 1.15:1, respectively, and in the amount of 1.0 wt.%. The reaction proceeded with constant stirring in an inert argon atmosphere at room temperature until latex was formed. For molecular imprinting in the surface layer, template cholesterol molecules were introduced after the latex formation and before the final granule formation. The completion of the formation of granules was carried out at a temperature of 37 °C in a water bath for 1 h. The content of the cholesterol template in the reaction mixtures was 2 mol.%, 4 mol.%, and 6 mol.%, calculated with respect to the weight of monomers.

#### 2.2.2. The Synthesis by Precipitation Polymerization in Solution

Polymer sorbents without the use of nanoparticles were synthesized similarly to [43] by free radical precipitation polymerization in n-propyl alcohol, hereafter referred to as “Method 2” (MIP-2).

The synthesis was carried out in a heat-resistant beaker equipped with a mechanical stirrer. HEMA and EGDMA were sequentially introduced into the n-propyl alcohol solution. The ratio and total concentration of monomers in solution were similar to Method 1. The polymerization reaction was initiated with a system of ammonium persulfate–ascorbic acid in the amount of 2.0 wt.% and the weight ratio of the system components of 1.15:1, previously dissolved in 1 mL of distilled water. The reaction proceeded with constant stirring in an inert argon atmosphere at room temperature until gelation. To complete polymerization, the mixture was heated in a water bath at 37 °C for 1 h. Synthesized polymers with a template content in the reaction mixtures of 2 mol.%, 4 mol.%, and 6 mol.% are hereinafter referred to as MIP-2-2, MIP-2-4, MIP-2-6, respectively.

Control polymers by Method 1 and Method 2 (CP-1 and CP-2) were synthesized without addition of template cholesterol molecules.

### 2.3. Purification of Synthesized Polymers

The synthesized polymer granules of both series were repeatedly washed with ethyl and n-propyl alcohol at room temperature. Extraction of cholesterol templates with n-propyl alcohol was carried out on imprinted granules in a Soxhlet apparatus at the boiling point of the solvent [43]. The cholesterol content in extracts was determined in accordance with the method for determining its concentration. Polymer granules, washed from the template, were repeatedly washed with distilled water. The removal of alcohol was monitored spectrophotometrically. To remove moisture, sorbents were kept in a drying oven (30–35 °C). After drying, granules were fractionated using molecular sieves. For further research, we used fractions of polymers with a granule size of 160–315 µm.

### 2.4. The Method for Determining the Concentration of Cholesterol

The content of cholesterol was determined using the enzymatic colorimetric test Cholesterol-Vital (St. Petersburg, Russia). Aliquots of 20 μL were taken from test tubes (or vials), 2 mL of the Cholesterol-Vital test reagent containing cholesterol esterase, peroxidase, and chromogenic substrates were added, then thermostated for 5 min at 37 °C [13,43]. Cholesterol was oxidized by atmospheric oxygen under action of cholesterol oxidase with the formation of an equimolar amount of hydrogen peroxide. Under action of peroxidase hydrogen peroxide oxidized chromogenic substrates (included in the Cholesterol-Vital test reagent) with the formation of a colored product that absorbed at λ = 500 nm. The color intensity was proportional to the cholesterol concentration in the sample.

The cholesterol concentration (mmol/L) in the sample was determined by the formula:

(2) $$C_{\mathrm{Chol}}=\frac{D_{\mathrm{Ex}}}{D_{C}}\times 5.17,$$

where *D*_Ex_—optical density of the analyzed solution, units of opt. dens.; *D*_C_—optical density of the calibrator, units of opt. dens.; and 5.17—concentration of cholesterol in the calibrator, mmol/L.

As the reference sample, 20 μL of n-propyl alcohol with 2 mL of the Cholesterol-Vital test reagent was used. The determination of optical densities of the experimental, *D*_Ex_, and calibration samples, *D*_C_, was carried out by means SF-256UVI spectrophotometer («LOMO» Photonica, Russia) at room temperature in a quartz cuvette with the optical path length of 10 mm (cuvette 1 cm × 1 cm) at the wavelength of 500 nm.

The confidence interval for determining cholesterol varied depending on its concentration in the solution: in the concentration range 0.50–3.00 mmol/L, the confidence interval was ±0.01 mmol/L; in the range of 3.00–5.17 mmol/L—± 0.07 mmol/L; in the range 5.17–12.00 mmol/L—± 0.20 mmol/L; in the range 12.00–17.00 mmol/L—± 0.30 mmol/L.

### 2.5. Physicochemical Properties of Polymers

Yields of synthesized polymers were calculated according to the formula:

(3) $$\mathrm{Yields}=\frac{m\times 100}{m_{\mathrm{theor}}}\%,$$

where *m*—the mass of the obtained polymer (g); and *m*_theor_—the theoretical mass of the polymer (g).

The bulk density, *ρ*_bulk_ (g/cm^3^), of synthesized polymers in the solvated state was determined by the gravimetric method by weighing 1 cm^3^ of the sorbent on an analytical balance, previously swollen in *n*-propyl alcohol (the alcohol was separated by centrifugation) [44]. This value includes the true density of sorbents, voids in the sorbent (porosity), and the space between particles.

The swelling coefficient, *K*_sw_, of the sorbent was determined as the ratio between the volume of the sorbent swollen in distilled water and n-propyl alcohol at temperatures of 25 °C and 37 °C, *V*_sw_ (mL), for 24 h until equilibrium was established, to the volume of dry sorbent, *V*_dry_ (mL) [44]:

(4) $$K_{\mathrm{sw}}=\frac{V_{\mathrm{sw}}}{V_{\mathrm{dry}}}.$$

The true density in the solvated state, *ρ*_true_ (g/cm^3^), was investigated by pycnometry at room temperature. To completely wet the pycnometer with a liquid, it was thoroughly washed with a chromium mixture, then with water, rinsed with distilled water, ethyl alcohol, and acetone, and dried. The empty pycnometer was then weighed, filled with the studied sorbent, and weighed again. The pycnometer containing the sorbent was filled with a liquid (n-propyl alcohol), first with ~0.5 volume to displace the air, and then to the calibration mark, and the mass of the pycnometer with sorbent and alcohol was determined. Refinement of the volume of the pycnometer was determined by weighing the liquid.

The true density of the sorbent was calculated by the equation:

(5) $$\rho_{\mathrm{true}}=\frac{m_{2}-m_{1}}{V_{\mathrm{pl}}-V_{l}}=\frac{\left(m_{2}-m_{1}\right)\cdot \rho_{l}}{m_{4}-m_{1}-\left(m_{3}-m_{2}\right)},$$

where *ρ*_l_—the density of the liquid in the pycnometer (density of n-propyl alcohol—0.8 g/cm^3^), g/cm^3^; *V*_pl_ and *V*_l_—adjusted volume of the liquid in the pycnometer and volume of the measured liquid, mL; and *m*_1_, *m*_2_, *m*_3_, and *m*_4_—the mass of the pycnometer, the mass of the pycnometer with sorbent, the mass of the pycnometer with sorbent and liquid, and the mass of the pycnometer with liquid, g. 

The total pore volume of the sorbent, determined in the swollen state (in n-propyl alcohol), *W*_0,sum_ (cm^3^/g), was calculated according to the equation:

(6) $$W_{0,\mathrm{sum}}=\frac{V_{s}/V_{p}-\alpha_{\mathrm{sw}}}{\left(1+\alpha_{\mathrm{sw}}\right)\cdot \rho_{\mathrm{true}}},$$

where *ρ*_true_—the true density of the polymer, g/cm^3^; *V*_s_—the volume of the solvent in the pores, mL; *V*_p_—the volume of the polymer, mL; and *α*_sw_—the degree of swelling.

The volume of the solvent in the pores, *V*_s_ (mL), was calculated by the formula [45]:

(7) $$V_{s}=\frac{m_{8}}{\rho_{l}},$$

where *m*_8_—the mass of the solvent in the pores of the swollen polymer, g:

*m*_8_ = *m*_5_ + *m*_6_ − *m*_7_, (8)

where *m*_5_—the mass of the liquid in the pycnometer, g:

*m*_5_ = *m*_4_ − *m*_1_, (9)

where *m*_6_—the mass of the swollen sorbent, g:

*m*_6_= *m*_2_ −*m*_1_, (10)

and *m*_7_—the mass of the swollen sorbent with liquid in the pycnometer, g:

*m*_7_ = *m*_3_ − *m*_1_, (11)

The volume of the polymer, *V*_p_ (mL), was determined in the swollen state without considering the solvent in the pores:

(12) $$V_{p}=\frac{m_{6}-m_{8}}{\rho_{\mathrm{true}}}.$$

The degree of swelling was calculated according to the formula:

(13) $$\alpha_{\mathrm{sw}}=\frac{m_{\mathrm{sw}}-m_{\mathrm{dry}}}{m_{\mathrm{dry}}}=K_{\mathrm{sw}}-1,$$

where *m*_dry_—the dry weight of the polymer, g; and *m*_sw_—the mass of the polymer in a swollen state, g.

The porosity of the sorbents in the solvated state, *ε* (%), was calculated by the formula:

(14) $$\varepsilon =1-\frac{\rho_{\mathrm{bulk}}}{\rho_{\mathrm{true}}}\times 100.$$

### 2.6. Scanning Electron Microscopy

Scanning electron microscopy (SEM) of the surface of sorbent samples was carried out by means of Carl Zeiss SUPRA-55VP electron microscope (Carl Zeiss AG, Berlin, Germany). To remove the charge and shield the incident beam from the charge accumulated in the bulk of the material, particles of copolymers were deposited on carbon tape NEM TAPE and a thin conductive carbon layer of ~10 nm was deposited on them.

### 2.7. Swelling Kinetics of Polymers

Study of kinetics of swelling of polymers synthesized by the Pickering emulsion polymerization and synthesized by polymerization in n-propyl alcohol was carried out in distilled water and n-propyl alcohol at temperatures of 25 °C and 37 °C.

Polymers with the fraction size of 160–315 μm were used for the study. Each tube with a sorbent (1 cm^3^) was carefully filled with 10 mL of distilled water or n-propyl alcohol. To avoid the formation of bubbles and uneven distribution of sorbent granules, tubes filled with liquid and polymers were gently tapped (if necessary, bubbles were carefully removed using a glass rod) and tubes were covered with lids. The change in the volume of polymer sorbents in distilled water and n-propyl alcohol was recorded at 25 °C and at 37 °C at regular intervals until equilibrium swelling was established. Experiments at 37 °C were carried out in a specialized (heating) cabinet.

### 2.8. The Equilibrium Sorption of Cholesterol on Synthesized Sorbents

Equilibrium sorption properties of polymer sorbents synthesized by Method 1 were studied in [45]. The current research studies the effect of the polymer synthesis method of their sorption properties and the affinity of sorption surfaces.

The study of the equilibrium of sorption was carried out under static conditions for 24 h at temperatures of 25 °C and 37 °C. Sorption equilibrium was studied using sorbent granules with a diameter of 160–315 μm. The alcoholic cholesterol solution was preliminarily prepared. The sample of cholesterol (433.1 mg) was dissolved in 70 mL of n-propyl alcohol, and a solution with a concentration of 16 mmol/L was obtained. Weighed portions of sorbents (20 mg) were placed in the penicillin vials. Sorbents were swollen in 0.1 mL of n-propyl alcohol for 20 min. In vials with swollen sorbents, 10 mL of the cholesterol solution with concentrations of 0.8, 1.0, 2.0, 4.0, 8.0, 10.0, 14.0, and 16.0 mmol/L was added and placed on a stirrer at a constant temperature. After a day, all vials were removed, aliquots of 20 μL were taken, and the equilibrium concentration of cholesterol was determined in accordance with the method described above.

The sorption capacity, *q*_eq_, of sorbents was calculated by the formula:

(15) $$q_{\mathrm{eq}}=\frac{\left(C_{0}-C_{\mathrm{eq}}\right)\times V}{m},$$

where *C*_0_ and *C*_eq_—the initial and equilibrium concentrations of cholesterol in the solution, respectively, mmol/L; *V*—the volume of the solution, mL; and *m*—the mass of the sorbent, g.

Further, curves of the dependence of the sorption capacity on the equilibrium concentration (sorption isotherms) were plotted.

### 2.9. Sorption Isotherm Models

The analysis of experimental data was carried out using theoretical models [46,47]: the Langmuir model [46,47,48,49,50], the Freundlich model [50,51,52], and the Brunauer–Emmett–Teller (B.E.T.) model [53,54,55].

The Langmuir model implies the sorption of solute molecules on a homogeneous sorption surface in the absence of interaction between sorbate molecules with the formation of a final monolayer and is described by the following equation:

(16) $$q_{\mathrm{eq}}=\frac{q_{\max}\times K_{L}\times C_{\mathrm{eq}}}{1+K_{L}\times C_{\mathrm{eq}}},$$

where *q*_max_—the maximum capacity of the monolayer (mmol/g); and *K*_L_—the distribution coefficient, the constant of sorption equilibrium for the formation of a monolayer (Langmuir parameter).

The Freundlich model assumes the binding of sorbate to energetically heterogeneous sorption sites:

(17) qeq=KF×Ceq1nf,

where *K*_F_—the Freundlich constant characterizing adsorption capacity or affinity of the sorbate to the sorbent; and 1/*n*_f_—index (factor) of heterogeneity. Usually, during sorption, the heterogeneity factor 1/*n*_f_ ranges from 0 to 1, tending to 0 as heterogeneity increases. A 1/*n*_f_ value of 1 indicates that the sorption surface is homogeneous. Freundlich’s model is applicable for both monolayer sorption and multilayer sorption.

In practice, the sorption process is often accompanied by the effect of multilayer binding of the sorbate. Brunauer, Emmett, and Teller proposed the B.E.T. sorption model, which makes it possible to describe isotherms of polymolecular sorption for the gas–solid system [56].

Ebadi et al. [53,54] adapted the B.E.T. model to a liquid–solid system:

(18) $$q_{\mathrm{eq}}=q_{\max}\times \frac{K_{L}\times C_{\mathrm{eq}}}{\left(1-K_{U}\times C_{\mathrm{eq}}\right)\times \left(1-K_{U}\times C_{\mathrm{eq}}+K_{L}\times C_{\mathrm{eq}}\right)},$$

where *K*_U_—the sorption equilibrium constant for upper layers (L/mmol); and *K*_L_—the constant of the sorption equilibrium for the first layer (L/mmol). In the absence of upper layers of solute molecules, i.e., *K*_U_ = 0, the B.E.T. model equation is converted to the Langmuir equation.

An additional assumption in the B.E.T. model, which differs from the Langmuir model, is the formation on the sorbent surface of “successive complexes” of sorption centers with one, two, three, etc., sorbate molecules. The authors of the model assumed that in all layers, except for the first one, it is mainly sorbate molecules that interact with one another.

To assess the applicability of theoretical models of sorption isotherms to obtained experimental data, functions of statistical errors calculated in OriginLab2019b were estimated: the corrected coefficient of determination (adj. *R*^2^) and the reduced criterion of goodness (*χ*^2^) as criteria for the convergence of experimental and theoretical data [57]. The measure *R*^2^ is used to analyze the degree of agreement of a theoretical model with experimental data; its value varies from 0 to 1. If the values of *R*^2^ tend to 1, then the theoretical models are most consistent with the experimental curves. For experimental values of *q*_eq_ comparable to the theoretical values of *q*_eq_, *χ*^2^ is close to 0. Therefore, the most appropriate model will be the model, when approximated, the least value of *χ*^2^ will be observed, and the value of *R*^2^ will tend to 1.

**Figure 1:**
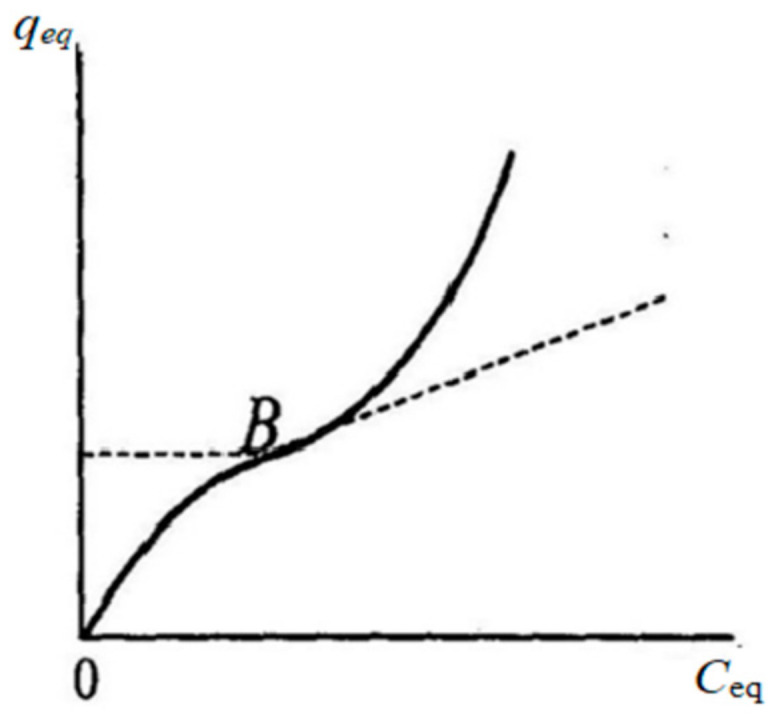
Determination of the capacity of a monolayer using the Brunauer point (point *B*) [59].

To calculate the sorption surface area available for binding of the target molecule, the *q*_max_ value in the case of correspondence of experimental data to the Langmuir and B.E.T. models was determined from these models. In case of unsatisfactory description of experimental data by theoretical models, the *q*_max_ value was determined graphically using the Brunauer point (point B) [58,59]. (Figure 1).

The area of the available sorption surface, *S*_SA_ (m^2^/g), for a cholesterol molecule in the “liquid–solid” system was calculated similarly to [23,45] according to the following equation:

(19) $$S_{\mathrm{SA}}=S_{\mathrm{mol}}\times N_{\mathrm{mol}},$$

where *S*_mol_—the area of a cholesterol molecule (0.56 nm^2^) [60], and *N*_mol_—the number of sorbate molecules (mol):

(20) $$N_{\mathrm{mol}}=N_{A}\times q_{\max},$$

where *N*_A_—the Avogadro number (6.022 × 10^23^ mol^−1^), and *q*_max_—the maximum capacity of the monolayer (mmol/g).

### 2.10. The Dynamic Sorption of Cholesterol

Experiments on the dynamics of sorption were carried out according to [61].

The dynamic sorption of cholesterol was carried out on glass columns with the inner diameter of 10 mm, filled with sorbents CP-1, MIP-1-2, MIP-1-4, and MIP-1-6. Sorption columns were preliminarily washed with a chromium mixture, then with water, rinsed with distilled water, and dried. The column was fixed on a stand strictly vertically and filled with pre-swollen sorbent granules, and then washed with n-propanol. An alcohol solution of cholesterol was preliminarily prepared. For this, the weighed portion of cholesterol (742.44 mg) was dissolved in 120 mL of n-propyl alcohol, and a solution with the initial concentration of 16 mmol/L was obtained. The solution with the test substance was fed into the column using a peristaltic pump (NP-1M, Russia).

The study of the influence of the mobile phase flow rate on the dynamics of sorption of cholesterol was carried out on columns with a sorption layer height (H) equal to 3.0 cm, at rates equal to 0.25 mL·min^−1^ and 0.5 mL·min^−1^. Samples (0.1 mL, 0.2 mL) at the column outlet were collected at regular intervals. After each dynamic sorption experiment, columns were washed with n-propyl alcohol. The control of the removal of cholesterol was carried out using the enzymatic colorimetric test Cholesterol-Vital.

Experiments to study the effect of H on the sorption of cholesterol under dynamic conditions were carried out with a constant flow rate equal to 0.25 mL·min^−1^, and with H equal to 3.0 cm and 4.5 cm.

Frontal dynamic curves were plotted in the coordinates *C*/*C*_0_ vs. *V*, where *C* and *C*_0_—cholesterol concentrations in the eluate and in the initial solution, respectively, mmol/L; and *V*—the volume of the eluate, mL.

The amount of cholesterol (mmol) introduced into the sorption column before saturation of the sorbent was calculated according to the formula:

(21) $$Q_{i}=C_{0}\times V_{i},$$

where *V*_i_—the volume of the initial cholesterol solution that was passed through the column until the sorbent was saturated.

The amount of cholesterol (mmol) in solution after sorption was calculated according to the formula:

(22) $$Q_{\mathrm{sol}}=\int_{0}^{V_{i}}C\;dV.$$

Then the sorbed amount of cholesterol (mmol) was calculated according to the formula:

(23) $$Q_{\mathrm{sorb}}=Q_{i}-Q_{\mathrm{sol}},$$

Equilibrium dynamic sorption capacities were calculated (mmol/mL) per 1 mL of sorbent according to the formula:

(24) $$q_{\mathrm{dyn}}=\frac{Q_{\mathrm{sorb}}}{V_{\mathrm{sorb}}},$$

where *V*_sorb_—the volume of the swollen sorbent in the column (mL).

The extraction degree of cholesterol (%) from an alcoholic solution was calculated by the formula:

(25) $$R=\frac{Q_{\mathrm{sorb}}}{Q_{i}}\times 100.$$

The imprinting factor was calculated according to the formula:

(26) $$IF=\frac{q_{\mathrm{MIP}}}{q_{\mathrm{CP}}},$$

where *q*_MIP_ and *q*_CP_—equilibrium dynamic sorption capacities of MIP and CP, respectively.

### 2.11. The Dynamic Sorption of Cholic Acid

**Figure 2:**
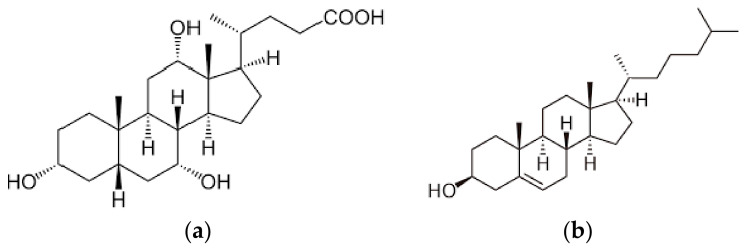
Structural formula of cholic acid (**a**) and cholesterol (**b**).

To study the selectivity of sorbents we implemented the sorption of cholic acid under optimal conditions for sorption of cholesterol. Cholic acid has the structural analogue of cholesterol (Figure 2).

The dynamic sorption of cholic acid was carried out on glass columns with an inner diameter of 10 mm, filled with CP-1 and MIP-1-6 sorbents. The preparation of sorption columns and equipment was carried out according to the procedure for conducting experiments on the sorption of cholesterol under dynamic conditions (Section 2.10). The alcoholic solution of cholic acid with an initial concentration of 16 mmol/L was preliminarily prepared. For this, a weighed portion of cholic acid (980.6 mg) was dissolved in 150 mL of n-propyl alcohol.

Experiments on the study of selectivity were carried out by means of CP-1 and MIP-1-6 columns with height (H) equal to 3.0 cm, at the flow rate equal 0.25 mL·min^−1^. Samples (0.2 mL) at the exit from the column were collected at regular intervals.

After each dynamic sorption experiment, the columns were washed with n-propyl alcohol. Control of washing from cholic acid was carried out spectrophotometrically at λ = 212 nm.

Frontal dynamic curves were plotted in coordinates *C*/*C*_0_ vs. *V*. The amount of cholic acid (mmol) introduced into the sorption column before saturation of the sorbent was calculated according to Formula (21). The amount of sorbed cholic acid (mmol) was calculated according to Equation (23), and *q*_din_ cholic acid according to (24).

The sorption selectivity coefficient was calculated by the Equation:

(27) $$\alpha =\frac{q_{\mathrm{Cholest}}}{q_{\mathrm{ChA}}},$$

where $q_{\mathrm{Cholest}}$ and $q_{\mathrm{ChA}}$—equilibrium dynamic sorption capacities of sorbents related to cholesterol and cholic acid, respectively.

### 2.12. Determination of Cholic Acid Concentration

To determine the concentration of cholic acid, preliminarily constructed calibration curves were used at the wavelength of 212 nm, which were linear from concentrations of 0.06 mmol/L to 2.48 mmol/L. To determine the concentration of cholic acid according to the calibration curve, samples (0.2 mL each) collected during the experiment on the dynamics of sorption were diluted by 10 times (up to 2 mL). Determination of optical densities of experimental samples was carried out by means of the spectrophotometer SF-256UVI (LOMO Photonica, Russia) at room temperature in the quartz cuvette with the optical path length of 10 mm (cuvette 1 cm × 1 cm) at the wavelength of 212 nm.

## 3. Results

### 3.1. Synthesis of Polymer Sorbents

Polymers based on 2-HEMA and EGDMA surface-imprinted with cholesterol molecules were synthesized by Pickering emulsion polymerization and precipitation polymerization in n-propyl alcohol for selective sorption of cholesterol.

The efficiency of polymerization was evaluated by the completeness of yields of polymers (Table 1). An increase in the amount of cholesterol introduced during the synthesis led to an increase in yields of polymer sorbents synthesized by both methods of synthesis.

### 3.2. The Morphology of Synthesized Polymers

**Figure 3:**
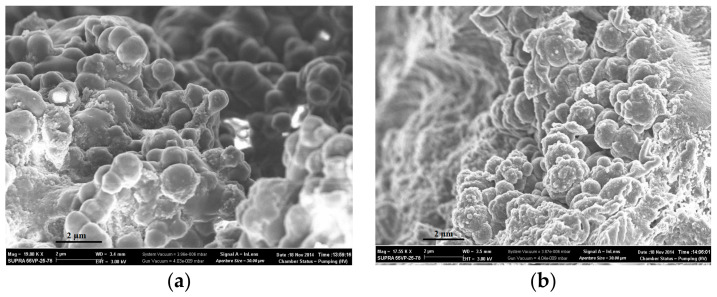
SEM of the surfaces of sorbents: network CP-1 (**a**) and network MIP-1-6 (**b**).

**Figure 4:**
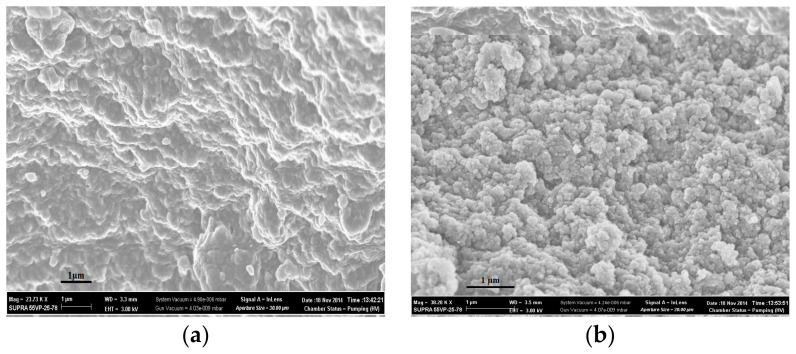
SEM of the surfaces of sorbents: network CP-2 (**a**) and network MIP-2-6 (**b**).

The morphology of polymers obtained by both synthesis methods was investigated using scanning electron microscopy (Figure 3 and Figure 4). Sorbents CP-1 and MIP-1-6 were synthesized in the form of spherical granules, which were microglobules cross-linked with each other (Figure 3a,b). The formation of granules was due to the method of the polymer synthesis by Pickering emulsion polymerization: when carrying out the polymerization reaction in Pickering emulsions, dispersed polymer-monomer droplets began to stick together, which leads to the formation of cross-linked microglobules.

The surfaces of sorbents obtained by Method 2 (Figure 4) were more uniform in comparison with the surfaces of sorbents obtained by Method 1. When carrying out precipitation polymerization in solution, the formation of polymer granules was carried out only by stirring the mixture. Polymer particles having reached a certain size, in our case ≥ 0.5 cm, during polymerization, fell out of the solution in the form of separate granules, which were subsequently subjected to crushing and fractionation.

In addition, the scanning electron microscopy showed that the addition of 6 mol.% cholesterol into the polymerization mixture led to the segregation of the surface of synthesized polymer granules.

### 3.3. Kinetics of Swelling of Polymers

**Figure 5:**
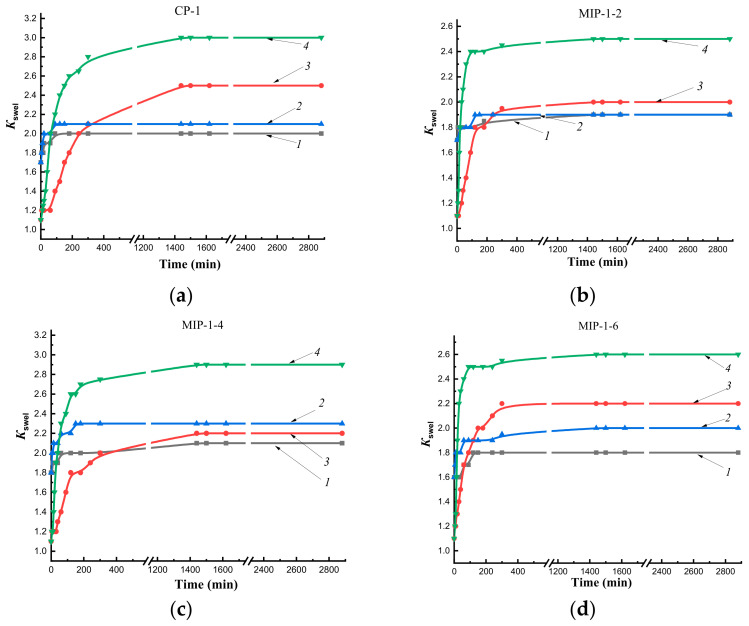
Kinetics of swelling sorbents obtained by Method 1 in water and n-propyl alcohol at temperatures of 25 °C and 37 °C: CP-1 (**a**), MIP-1-2 (**b**), MIP-1- 4 (**c**), MIP-1-6 (**d**); *1*—in water at 25 °C; *2*—in water at 37 °C; *3*—in n-propyl alcohol at 25 °C; *4*—in n-propyl alcohol at 37 °C.

On kinetic curves of swelling of polymer sorbents obtained by synthesis Method 1 in water at temperatures of 25 °C and 37 °C, the formation of a short plateau preceding the achievement of equilibrium swelling was observed (Figure 5), which indicated difficult diffusion of the solvent into the polymer networks. This was probably due to the formation of a shell of sorbent granules containing Se/PVP nanocomplexes.

**Figure 6:**
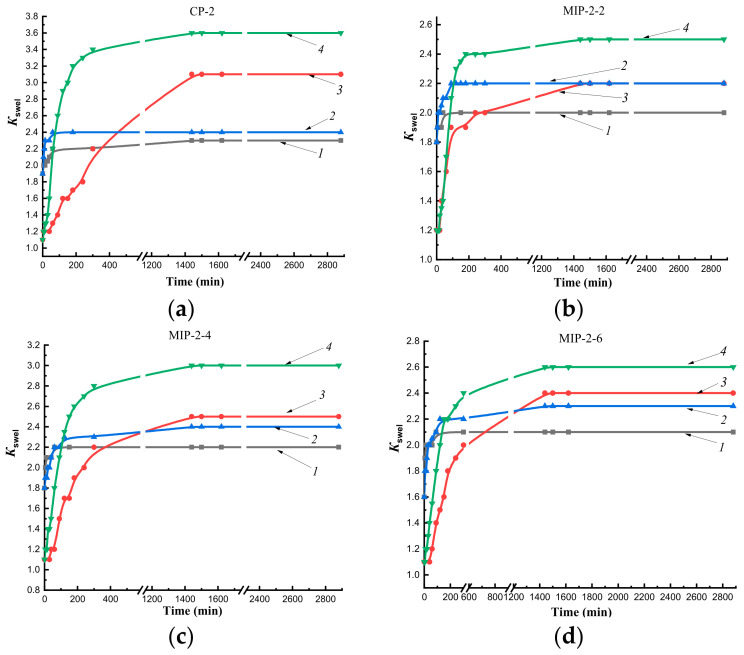
Kinetics of swelling sorbents obtained by Method 1 in water and n-propyl alcohol at temperatures of 25 °C and 37 °C: CP-2 (**a**), MIP-2-2 (**b**), MIP-2-4 (**c**), MIP-2-6 (**d**); *1*—in water at 25 °C; *2*—in water at 37 °C; *3*—in n-propyl alcohol at 25 °C; *4*—in n-propyl alcohol at 37 °C.

In initial sections of curves describing the swelling of MIP-1 (Figure 5b–d) in n-propyl alcohol, a sharp rise in the curve was observed, which characterized the rapid kinetics of swelling in comparison with CP-1 (Figure 5a) and MIP-2 (Figure 6b–d). Imprinting of polymer networks improved the diffusion of the solvent deep into the MIP-1 granules as compared to CP-1 at both temperatures. The synthesis of MIPs by Pickering emulsion polymerization led to improvement in the diffusion of n-propyl alcohol in comparison with imprinted polymer sorbents synthesized in a solvent.

The swelling kinetics of MIP-2 in n-propyl alcohol at a temperature of 25 °C improved compared to CP-2 (Figure 6). In the figures, this is displayed by a slow gentle rise in the curve describing the swelling of CP-2 (Figure 6a), whereas in MIPs, there is a sharp rise in the initial section of the kinetic swelling curve (Figure 6b–d). Thus, the addition of the cholesterol template to the polymerization mixtures led to an improvement in the diffusion of alcohol into the depths of polymer granules at a temperature of 25 °C as compared to CP-2.

At a temperature of 37 °C in n-propyl alcohol, there was a rapid increase in volumes of CP-2, MIP-2-4, and MIP-2-6 as the solvent diffused deep into polymer networks compared to swelling at a temperature of 25 °C (Figure 6c,d), whereas the increase in the volume of MIP-2-2 was carried out at the same rate up to 50 min both at room temperature and at 37 °C.

Thus, the study of the kinetics of swelling in water at temperatures of 25 °C and 37 °C showed the presence of a short plateau that indicated delay of solvent diffusion into the interior of polymer networks due to the effect of a shell of sorbent granules obtained by Method 1. The addition of the cholesterol template into polymerization mixtures improved diffusion of the alcohol into the depths of MIP granules obtained by both synthesis methods (at both temperatures for MIP-1, at room temperature for MIP-2) compared to CP. The synthesis of sorbents by Pickering emulsion polymerization led to an improvement in the diffusion of n-propyl alcohol into polymer networks as compared to polymer sorbents synthesized by precipitation polymerization in solution.

### 3.4. Physicochemical Properties of Polymers

The complementarity of sorption centers was created under conditions of formation of the polymer network and depended on the rigidity and structural stability of the polymer network. The study of the swelling of synthesized sorbents, obtained by Method 1, in solvents of different natures (n-propyl alcohol, water) at room temperature showed that the nature of the solvent had practically no effect on stretching of the polymer network of MIP-1-2 and MIP-1-4 as compared to with CP-1 and MIP-1-6 (Table 2). This indicated a more homogeneous structure of their networks.

During the swelling of polymer sorbents of Method 2 under similar conditions, it was shown that the nature of the solvent had the least effect on stretching of the polymer network MIP-2-2 as compared to MIP-2-4 and MIP-2-6, which also indicated a more homogeneous network structure. At the same time, it was shown that the addition of cholesterol as template molecules in the synthesis of imprinted polymers promoted the formation of more uniform polymer networks as compared to CP-2.

An increase in temperature upon swelling in water to the synthesis temperature did not have a significant effect on networks CP-1, MIP-1-2, and CP-2 that indicated their structural stability. The addition of a larger amount of template to polymerization mixtures led to a decrease in the structural stability of polymer networks.

At the same time, a decrease in the swelling coefficients of imprinted sorbents of both series, regardless of the nature of the solvent, indicated more rigidly crosslinked homogeneous networks as compared to CP. However, networks were not truly rigid (swelling coefficients did not tend to 1), therefore, surface areas and total pore volumes determined in the dry state will not correspond to these parameters in the swollen state. Thus, characteristics of polymer sorbents, which will be further used in liquid chromatography, are best determined in a solvated state.

An increase in values of porosities in the solvated state, *ε*, of imprinted polymers of Method 1 in comparison with CP-1 indicated formation of additional pores due to the addition of template cholesterol molecules (Table 2). Values of total pore volumes determined in the swollen state, *W*_0,sum_, also increased with an increase in the amount of the introduced template during the synthesis. Consequently, an increase in the total pore volumes was due to the creation of imprint sites. However, MIP-1-4 was out of the general trend. Apparently, this is due to formation of the network capable of greater stretching of the polymer network by solvation with solvent molecules in comparison with MIP-1-2 and MIP-1-6, that was also reflected in the porosity and the total pore volume determined in the solvent.

The addition of cholesterol as template molecules to polymerization mixtures during the synthesis of MIP-2 led to a decrease in values of *ε* and *W*_0,sum_, compared to CP-2. This was due to the formation of more rigidly crosslinked polymer networks, in contrast to their control analog. It should be noted that in a series of MIPs of Method 2, the highest values of *ε* and *W*_0,sum_ were observed in MIP-2-4. Apparently, the modification of the HEMA-EGDMA polymer network with 4 mol.% cholesterol led to the formation of a more extensible and less rigidly crosslinked polymer network compared to MIP-2-2 and MIP-2-6.

Values of *W*_0,sum_ of MIP-1-2 and MIP-1-6 slightly exceeded *W*_0,sum_ of MIP-2-2 and MIP-2-6, and values of the total pore volume of MIP-4 of both series were equal. Consequently, the synthesis method had no significant effect on the total pore volume of synthesized polymers upon modification of the polymer network with 4 mol.% cholesterol.

Thus, the synthesis of sorbents by Pickering emulsion polymerization led to formation of polymer networks with higher porosity compared to polymer sorbents synthesized in solution.

### 3.5. Equilibrium Sorption of Cholesterol by Synthesized Sorbents

The study of the equilibrium sorption of cholesterol by surface-imprinted sorbents will make it possible to establish the nature of the binding of solute molecules to sorption surfaces of polymers. Different mechanisms of binding of molecules of the sorbed substance to the polymer surface lead to a variety of types of sorption isotherms. Based on the type of equilibrium sorption isotherms according to the Giles’ classification [62], it is possible to reveal the affinity of the sorption surface for the target molecule.

**Figure 7:**
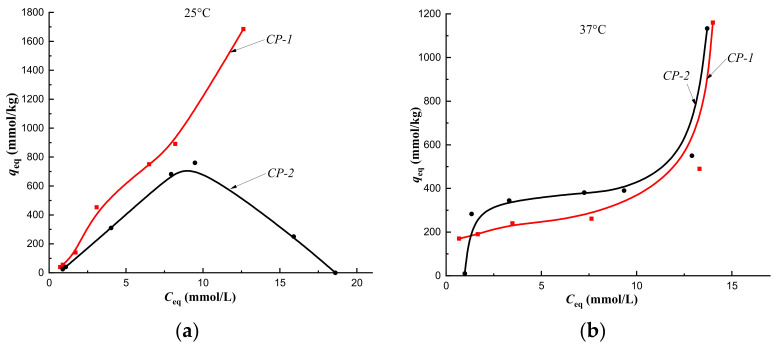
Isotherms of sorption of cholesterol on CP-1 and CP-2 at a temperature of 25 °C (**a**) and at a temperature of 37 °C (**b**) (fraction 160–315 μm).

**Figure 8:**
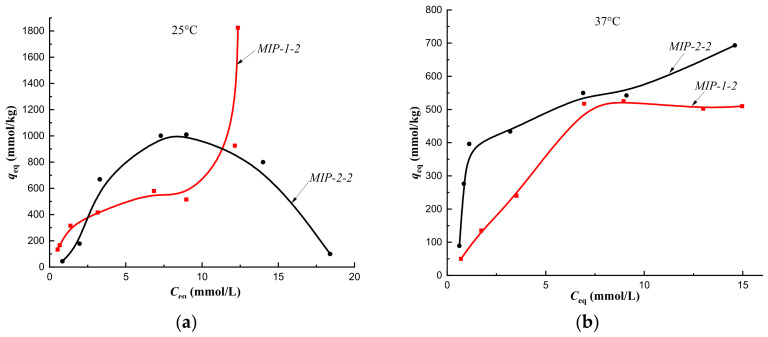
Isotherms of sorption of cholesterol on MIP-1-2 and MIP-2-2 at a temperature of 25 °C (**a**) and at a temperature of 37 °C (**b**) (fraction 160–315 μm).

**Figure 9:**
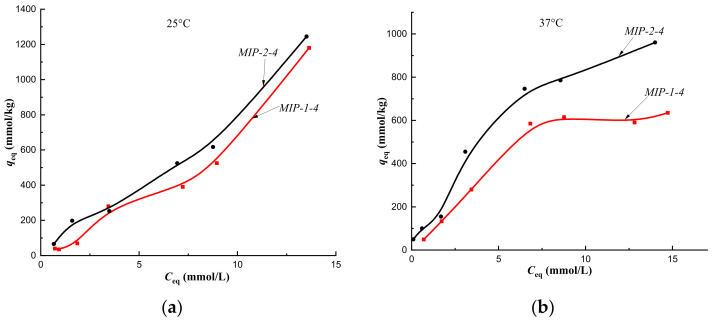
Isotherms of sorption of cholesterol on MIP-1-4 and MIP-2-4 at a temperature of 25 °C (**a**) and at a temperature of 37 °C (**b**) (fraction 160–315 μm).

**Figure 10:**
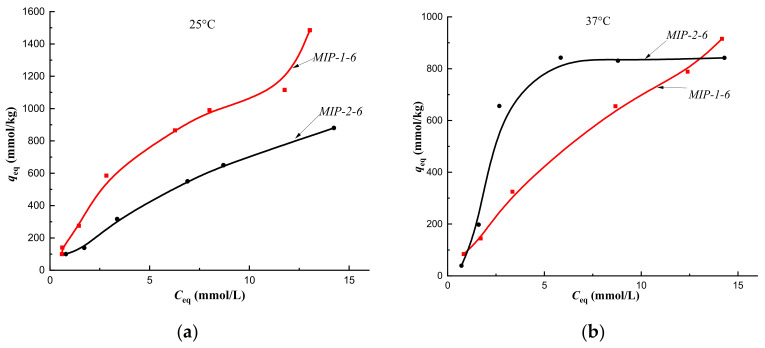
Isotherms of sorption of cholesterol on MIP-1-6 and MIP-2-6 at a temperature of 25 °C (**a**) and at a temperature of 37 °C (**b**) (fraction 160–315 μm).

Cholesterol sorption isotherms obtained at 25 °C on CP-1 and MIPs of Method 1 indicated the implementation of multilayer binding (Figure 7a, Figure 8a, Figure 9a and Figure 10a, red curves). At the same time, at a temperature of 37 °C, monolayer sorption was realized on MIPs, and multilayer sorption was observed only on CP-1 (Figure 7b, Figure 8b, Figure 9b and Figure 10b, red curves).

The equilibrium sorption of cholesterol from a model one-component solution on CP-2 and MIP-2-2 at 25 °C was described by isotherms with a maximum, which was due to the competitive sorption of solute molecules with a solvent (Figure 7a and Figure 8a, black curves). At 25 °C, multilayer sorption was realized on MIP-2-4 and monolayer sorption on MIP-2-6 (Figure 9a and Figure 10a, black curves). At 37 °C, sorption on imprinted sorbents MIP-2-2 and MIP-2-6 was characterized as a monolayer (Figure 8b and Figure 10b, black curves), and on CP-2 and MIP-2-4 as a multilayer (Figure 7b and Figure 9b, black curves).

According to Giles’ classification, cholesterol sorption isotherms obtained on CP-1 and CP-2 at 25 °C belong to the S type isotherm. This indicated a low affinity of the sorption surface to the solute (Figure 7a). At 37 °C the cholesterol sorption isotherms belonged to the H type isotherm, which indicated a high affinity of the sorption surface (Figure 7b). Change in the type of sorption isotherms from S to H, i.e., from a weak affinity of the sorption surface to a strong one, was due, apparently, to changing the orientation of the cholesterol molecule upon binding to the polymer network at a sorption temperature of 37 °C. As a result, a strong affinity of the hydrophobic surface of the sorbent for the hydrophobic part of the cholesterol molecule was observed, and its part with a hydroxyl group formed a sorption surface for binding the next layer of the solute to a greater extent. This was reflected by the presence of the plateau on curves of sorption isotherms at 37 °C (Figure 7b) compared to curves at a temperature of 25 °C (Figure 7a). Thus, the formation of the HEMA-EGDMA polymer in the presence of Se/PVP nanocomplexes did not lead to a significant change in the affinity of the sorption surface for cholesterol molecules in comparison with the sorption surface of the polymer synthesized in the absence of nanocomplexes.

At a temperature of 25 °C, the cholesterol sorption isotherm on MIP-2-2 belonged to type S, which indicated a low affinity of the sorption surface, and on MIP-1-2 it belonged to type L (Langmuir-type isotherm) (Figure 8a). Therefore, the addition of 2 mol.% cholesterol template to the polymerization mixture during the synthesis of the polymer sorbent by Pickering emulsion polymerization led to an increase the affinity of the sorption surface of HEMA-EGDMA to solute molecules.

However, upon modification of the polymer network with 4 mol.% cholesterol, a decrease in the affinity of the sorption surfaces of both MIP-1-4 and MIP-2-4 was observed (Figure 7a), as sorption isotherms belonged to the S type. This was probably due to the sorption of cholesterol on defective binding sites.

Addition of a template in the amount of 6 mol.% during synthesis of MIP-1-6 and MIP-2-6 led to a significant increase in the affinity of polymer surfaces for cholesterol (sorption isotherms belonged to the H type) (Figure 10a).

At a temperature of 37 °C (corresponding to the synthesis temperature), sorption isotherms obtained on MIP-2-2 and MIP-2-4 belonged to the H type, and on MIP-1-2 and MIP-1-4–to C type isotherms (Figure 8b; Figure 9b). Consequently, the equilibrium sorption of the target metabolite at the temperature of the formation of imprint sites (the temperature at which imprint sites were complementary to cholesterol molecules) led to an increase in the affinity of the sorption surface of polymer sorbents of Method 2 and an improvement in the availability of sorption centers of polymer sorbents of Method 1. In addition, the synthesis of MIPs by Pickering emulsion polymerization provided better accessibility of sorption centers MIP-1-2 and MIP-1-4 compared to MIP-2-2 and MIP-2-4.

The sorption isotherm obtained on MIP-2-6 at a temperature of 37 °C is described by the S type, which indicated a low affinity of the sorption surface, whereas on MIP-1-6 it belonged to type H; therefore, the implementation the synthesis of polymers by Pickering emulsion polymerization led to an increase in the affinity of the sorption surface of MIP-1-6 to the target cholesterol molecule (Figure 10b).

Thus, the addition of 6 mol.% cholesterol to the polymerization mixture promoted formation of sorption surfaces of Method 1 and Method 2 polymers with high affinity for cholesterol molecules at a sorption temperature of 25 °C. It has been established that the synthesis of polymer sorbents by Pickering emulsion polymerization in the presence of selenium nanoparticles leads to the formation of the MIP-1-6 sorption surface with a high affinity for cholesterol both at the synthesis temperature and at room temperature. It was also shown that synthesis of MIPs by Pickering emulsion polymerization led to an improvement in the availability of sorption centers MIP-1-2 and MIP-1-4 compared to MIP-2-2 and MIP-2-4. At the same time, the improvement of the accessibility of sorption centers MIP-1-2 and MIP-1-4 in comparison with CP-1 was facilitated by the implementation of the synthesis of surface-imprinted sorbents and the sorption of the target metabolite at the same temperature (temperature of the formation of imprint sites).

### 3.6. Analysis of Experimental Sorption Isotherms by Theoretical Models

The aim of studying the correlation of experimental sorption isotherms to generally accepted theoretical models is, first of all, to obtain information on the distribution of sorbent molecules between phases, the nature of the interaction between the sorbate and the sorbent, the capacity of the monolayer and the maximum sorption capacity *q*_max_, as well as the homogeneity of sorption centers. This information is necessary to optimize the sorption process of extracting the target substance from the multicomponent system.

#### 3.6.1. Langmuir Model

Experimental data on isotherms of cholesterol sorption by polymer sorbents (Method 1) at a temperature of 25 °C were satisfactorily described by the Langmuir model up to an equilibrium cholesterol concentration of 9.0 mmol/L upon sorption on MIP-1-2 and 11.8 mmol/L upon sorption on MIP-1-6 (Table 3).

This indicated the formation of a complete sorbate monolayer on the surface of these polymers at the given solute concentrations. However, during the sorption of cholesterol on CP-1 and MIP-1-4, theoretical curves of the Langmuir model did not correspond to the experimental points. Consequently, the experimental data on the sorption of cholesterol on CP-1 and MIP-1-4 were not described by the Langmuir equation in the entire concentration range.

Experimental data on isotherms of cholesterol sorption by polymer sorbents HEMA-EGDMA Method 2 at a temperature of 25 °C were satisfactorily described by the Langmuir model up to the equilibrium cholesterol concentration of 8.8 mmol/L during sorption on MIP-2-4 and in the entire concentration range during sorption on MIP-2-6 (Table 3), which indicated the formation of a monolayer. However, theoretical curves of the Langmuir model did not correspond to the experimental points for the sorption of cholesterol on CP-2 and MIP-2-2. Apparently, this could be due to the competition of solute molecules with solvent molecules for sorption centers, as experimental sorption isotherms have a maximum, which indicated competitive sorption according to the Giles’ classification. Furthermore, sorption isotherms belonged to the S type, which indicated a weak affinity of sorption surfaces of CP-2 and MIP-2-2 to cholesterol.

At 37 °C, the approximation of sorption isotherms on CP-1 by the Langmuir model was carried out up to the equilibrium concentration of 7.6 mmol/L, on MIP-1-6–up to 12.4 mmol/L, and indicated monolayer sorption. Sorption on MIP-1-2 and MIP-1-4 did not correspond to the Langmuir model because the theoretical curve did not reach a plateau in the region of experimental values, and sorption isotherms corresponded to type C. Experimental data on the sorption of cholesterol on MIP-2-4 at 37 °C satisfied the Langmuir model in the entire concentration range, which indicated monolayer sorption. Isotherms of cholesterol sorption on CP-2, MIP-2-2, and MIP-2-6 at a temperature of 37 °C did not satisfy the Langmuir model, as theoretical curves did not correspond to experimental points.

It was shown that implementation of the polymers synthesis by Pickering emulsion polymerization led to implementation of the monolayer sorption on CP-1 and MIP-1-6 at a temperature of 37 °C, corresponding to the synthesis temperature, whereas the implementation of the synthesis of polymers in a solvent was only on MIP-2-4. Thus, the implementation of the synthesis of the polymer sorbent by Pickering emulsion polymerization with the addition of 6 mol.% template at sorption temperatures of 25 °C and 37 °C led to formation of a complete sorbate monolayer.

#### 3.6.2. Freundlich Model

Experimental data of cholesterol sorption isotherms at 25 °C were satisfactorily described by the Freundlich model up to the equilibrium concentration: for CP-1—1.7 mmol/L, for MIP-1-2—1.4 mmol/L, for MIP-1-4—1.9 mmol/ L, and for MIP-1-6—2.8 mmol/ L; at 37 °C to the equilibrium concentration: CP-1—7.6 mmol/L, for MIP-1-2—7 mmol/L, for MIP-1-4—6.8 mmol/L, and for MIP-1-6—3.4 mmol/L (Table 4). Experimental data of cholesterol sorption isotherms were satisfactorily described by this model at a temperature of 25 °C to the equilibrium concentration: for CP-2—4 mmol/L, for MIP-2-2—3.3 mmol/L, for MIP-2-4—8.8 mmol/L, and for MIP-2-6—8.7 mmol/L; for MIP-2-2—1.1 mmol/L, for MIP-2-4—1.7 mmol/L, and for MIP-2-6—2.7 mmol/L (Table 4). The isotherm of cholesterol sorption on CP-2 at 37 °C did not satisfy the Freundlich model.

When approximating experimental data on the sorption of cholesterol at 25 °C on CP-1, CP-2, and MIP-2-2, the value of the heterogeneity factor 1/*n*_f_ was greater than 1, which indicated physical interactions between sorbate molecules, which were necessary for binding to the sorption surface. This may be due to the joint sorption of cholesterol molecules, as sorption isotherms belonged to the S type according to Giles’ classification. This also indicated the low affinity of sorption surfaces CP-1, CP-2, and MIP-2-2. At the same time, small *K*_F_ values confirmed the low affinity of their sorption surfaces for solute molecules. For CP-1 at a sorption temperature of 37 °C, an increase in *K*_F_ as compared to *K*_F_ at a sorption temperature of 25 °C indicated an increase in the affinity of the sorption surface, which was consistent with a change in the type of sorption isotherm from S to H. At the same time, the value of 1/*n*_f_ tends to 0, which indicated the heterogeneous sorption surface of CP-1. With an increase in the swelling of the polymer network with increasing temperature (the specific swelling increased from 2.5 to 3.0), the availability of carbonyl groups in the polymer network, which interacted with the hydroxyl group of cholesterol, improved. As a result, at a temperature of 37 °C, the polymer network began to possess a heterogeneous surface, namely, regions of the polymer chain capable of hydrophobic interactions, and carbonyl groups capable of forming hydrogen bonds with cholesterol molecules. In turn, the isotherm of cholesterol sorption on CP-2 at 37 °C did not satisfy Freundlich’s model. The 1/*n*_f_ value characterizing the sorption of cholesterol on MIP-2-2 was greater than 1, which also indicated binding to the sorption surface through additional physical interactions.

The sorption of cholesterol on MIP-1-2 at 25 °C was carried out on a heterogeneous surface (1/*n*_f_ < 1). However, sorption at a temperature corresponding to the temperature of the formation of imprint sites (37 °C) occurred on a homogeneous surface (1/*n*_f_ = 1). Consequently, imprint sites at a temperature inappropriate to the temperature of their formation lose complementarity to target molecules. This also affected the sorption of cholesterol on MIP-1-4 at a temperature of 25 °C (1/*n*_f_ < 1). However, at a sorption temperature of 37 °C, 1/*n*_f_ > 1. Consequently, an increase in the amount of the added template to 4 mol.% led to creation of sites that required additional physical interactions for sorptive binding. Sorption on MIP-1-6 at temperatures of 25 °C and 37 °C was also carried out on a homogeneous surface, requiring additional physical interactions between sorbed molecules. The 1/*n*_f_ values characterizing the sorption of cholesterol on MIP-2-4 and MIP-2-6 at 25 °C indicated formation of a more heterogeneous sorbent surface (1/*n*_f_ <1) compared to CP-2 and MIP-2-2. An increase in temperature to 37 °C led to stretching of the polymer network of MIP-2-4 and MIP-2-6 (Table 4), which was reflected in a change in the nature of cholesterol binding. In the first case, the 1/*n*_f_ value indicated sorption on a more heterogeneous surface compared to sorption at a temperature of 25 °C, and in the second case, the 1/*n*_f_ value indicated sorption on a surface with weak affinity (S type) with the need for additional physical interactions.

The highest *K*_F_ value for the sorption of cholesterol on MIP-1-2 at a temperature of 25 °C indicated the highest affinity of its sorption surface for cholesterol molecules. This agreed with the types of sorption isotherms according to Giles’ classification: CP-1 and MIP-1-4 belonged to the S type, MIP-1-2 to the L type. During the cholesterol sorption at a temperature of 37 °C, corresponding to the synthesis temperature, the highest *K*_F_ value was observed for sorption on CP-1. This also correlated with the fact that the sorption isotherm of cholesterol on this sorbent belonged to the H type, indicating a strong affinity of the sorption surface. The C type from the Giles’ classification, which included sorption isotherms on MIP-1-2 and MIP-1-4, did not characterize the affinity of the sorption surface in any way, but indicated an improvement in the availability of sorption centers; therefore, it was impossible to correlate with *K*_F_. The *K*_F_ values for MIP-2-4 and MIP-2-6 at a temperature of 25 °C were equal to and greater than *K*_F_ values for CP-2 and MIP-2-2. This indicated a high affinity of their sorption surfaces for cholesterol molecules. In the case of MIP-2-6, this was confirmed by the H type of sorption isotherms according to the Giles’ classification. In the case of MIP-2-4, the sorption isotherm belonged to the S type, indicating a weak affinity of the sorption surface, but competitive sorption of cholesterol was carried out on CP-2 and MIP-2-2. Both of these facts could be reflected in such *K*_F_ values.

The *K*_F_ value for the sorption of cholesterol molecules on MIP-1-6 fell out of the general trend. Although at a temperature of 25 °C, *K*_F_ agreed with the H type of the sorption isotherm, its value was less than for *K*_F_ MIP-1-2, in which the sorption isotherm belonged to the L type, i.e., the sorption surface of MIP-1-6 according to Giles’ should have a high affinity. At a temperature of 37 °C, corresponding to the synthesis temperature, there was also no correlation with the type of isotherm. This may be because the Freundlich model was applicable to sorption on a heterogeneous surface, whereas MIP-1-6 had a homogeneous surface. At the same temperature, the low *K*_F_ value observed for the sorption of cholesterol on MIP-2-6 correlated with the S type of the sorption isotherm and also indicated a low affinity of the polymer network for cholesterol molecules.

It was shown that the binding of cholesterol at a temperature of 25 °C by sorption surfaces CP-1, CP-2, and MIP-2-2 and at a temperature of 37 °C MIP-1-4, MIP-2-2, and MIP-2-6 was carried out by means of additional physical interactions between sorbate molecules. At a temperature of 25 °C, the binding of the solute occurred on the heterogeneous surfaces of sorbents MIP-1-2 and MIP-1-4, MIP-2-4, and MIP-2-6 and at 37 °C, on CP-1 and MIP-2-4. The sorption of cholesterol occurred on the homogeneous sorption surface MIP-1-6, which had a high affinity for solute molecules at both temperatures, and at 37 °C–on MIP-1-2.

Thus, the synthesis of polymer sorbents by Pickering emulsion polymerization with the addition of 6 mol.% of a template led to the formation of the homogeneous sorption surface with a high affinity for cholesterol both at the synthesis temperature and at room temperature, and with the addition of 2 mol.% of the template, at a temperature corresponding to the temperature of the formation of imprint sites.

#### 3.6.3. Brunauer–Emmett–Teller Model

Experimental data on isotherms of cholesterol sorption at a temperature of 25 °C were satisfactorily described by the B.E.T. model for CP-1, and for sorption on MIP-1-2 and MIP-1-4 there was no complete agreement with the experimental data in the entire range of equilibrium concentrations (Table 5). This was due to the heterogeneity of the sorption surfaces of the MIPs.

Sorption on MIP-1-6 did not satisfy the B.E.T. model in the region of the inflection point corresponding to the formation of the second layer. Isotherms of cholesterol sorption on CP-1, MIP-1-2, and MIP-1-4 at 37 °C did not satisfy the B.E.T. model, as theoretical curves did not agree with experimental data. This was because sorption isotherms on CP-1 corresponded to the H type, and on MIP-1-2 and MIP-1-4–to the C type, as well as the heterogeneity of their sorption surfaces. For the sorption of cholesterol on MIP-1-6 at 37 °C, experimental data were well described by the B.E.T. equation and indicated multilayer sorption.

The *K*_L_ and *K*_U_ values (the equilibrium constant of sorption for the first layer and the equilibrium constant of sorption for the upper layers, respectively) determined from the B.E.T. model at 25 °C for CP-1 indicated stronger interaction of the sorption surface with sorbate molecules than the interaction of the sorbate-sorptive during the formation of a multilayer. The sorption isotherm on CP-1 did not satisfy the Langmuir model, but it did satisfy the B.E.T. model, which may indicate the beginning of the formation of a multilayer during the formation of a monolayer. In the case the sorption on MIP-1-6 at both temperatures, *K*_L_ and *K*_U_ values indicated a stronger sorbate-sorbent interaction as compared to sorbate-sorptive interactions.

Isotherms of cholesterol sorption at 25 °C on CP-2 and MIP-2-2 and at 37 °C on CP-2, MIP-2-2, and MIP-2-6 did not satisfy the B.E.T. model, as theoretical curves did not correspond to the experimental data. This was probably because sorption isotherms at 25 °C on CP-2 and MIP-2-2 belonged to the S type, and also described the competitive sorption of cholesterol; at a temperature of 37 °C on CP-2 and MIP-2-2, they corresponded to H type, and on MIP-2-6–to S type. Experimental data on isotherms of sorption of cholesterol by polymer sorbents Method 2 at a temperature of 25 °C were satisfactorily described by the B.E.T. model for MIP-2-4, which indicated multilayer sorption.

Despite the low affinity of the sorption surface of MIP-2-4 to the target molecule (S type), *K*_L_ and *K*_U_ values determined from the B.E.T. model at a temperature of 25 °C for MIP-2-4 indicated a stronger interaction of the sorption surface with sorbate molecules than the interaction of the sorbate-sorbate. Isotherms of cholesterol sorption by polymer sorbents were also satisfactorily described by the B.E.T. model at a temperature of 25 °C for MIP-2-6 and at a temperature of 37 °C for MIP-2-4. Values of constants characterizing the binding of upper layers in the multilayer, *K*_U_, were of the order of 10^−16^ (Table 5). This indicated the low affinity of the formed monolayer to solute molecules. At the same time, *K*_L_ and *q*_max_ values of MIP-2-6 corresponded to values obtained by approximation by the Langmuir model, which indicated monolayer sorption. In addition, *K*_L_ and *K*_U_ values both for the sorption of cholesterol at a temperature of 25 °C on MIP-2-6 and at a temperature of 37 °C on MIP-2-4 indicated a stronger interaction between sorbent and sorbate.

It was found that at a temperature of 25 °C the sorption of cholesterol on CP-1, MIP-1-6, MIP-2-4, and MIP-2-6 and at a temperature of 37 °C on MIP-1-6 and MIP-2-4 indicated a stronger interaction of sorption surfaces of polymers with sorbate molecules, in comparison with sorbate-sorptive interactions during the formation of a multilayer. Thus, the implementation of polymer synthesis by Pickering emulsion polymerization with the addition of 6 mol.% cholesterol template led to the formation of a sorption surface with a higher affinity for cholesterol molecules in comparison with the formed monolayer, i.e., compared to sorbate-sorptive interactions at both temperatures. This also correlated with the H type of sorption isotherms, indicating a high affinity of the sorption surface of MIP-1-6 for solute molecules.

### 3.7. The Area of the Available Sorption Surface

The main characteristic of polymer sorbents is a specific surface area. It is usually determined by the adsorption of N_2_, i.e., in the “gas–solid” system with subsequent approximation of experimental isotherms by the B.E.T. model. However, placing sorbents in a liquid leads to the solvation of the polymer network, as a result of which, in the case of non-rigidly cross-linked polymers, their chains are stretched. Consequently, the surface area of the polymer determined by the adsorption of N_2_ will be unreliable for a stretched (swollen in an alcohol solution) polymer network. Therefore, the determination of the surface area of polymers in the solvated state will reveal the sorption surface area available for the sorption of the target molecule in the “liquid–solid” system.

As *q*_max_ was determined from models of sorption isotherms or graphically from curves describing the saturation of sorption sites, in those cases where it was possible to determine *q*_max_, specific surface areas, *S*_SA_, were calculated (Table 6).

An increase in the amount of the added template in the polymerization mixture during the synthesis of sorbents of Method 1 led to an increase in *S*_SA_ both at a temperature of 25 °C and at a temperature of 37 °C, which corresponded to the synthesis temperature. In turn, for polymer sorbents of Method 2, with an increase in the amount of cholesterol template, an increase in *S*_SA_ values was observed only at a sorption temperature of 37 °C.

### 3.8. Dynamic Sorption of Cholesterol

Knowledge of structural features of sorbents, as well as equilibrium regularities of the sorption, is insufficient to develop an effective dynamic process for the selective sorption of the target component from a multicomponent mixture. As the target component migrates along the sorption column, its concentration profile is blurred under influence of kinetic factors. This leads to the need to implement conditions for the formation of sharp boundaries of concentration profiles of substances, as well as finding physicochemical properties of the sorbent-sorbate system, at which there is a transition from an equilibrium to a nonequilibrium dynamic mode of sorption. The solution of these problems is associated with the analysis of the regularities of mass transfer of the target component.

**Figure 11:**
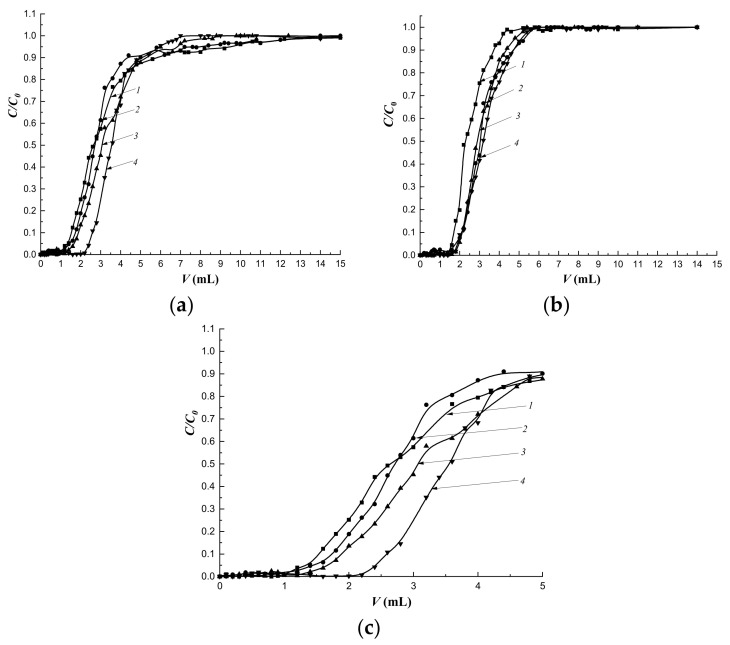
Dynamics of cholesterol sorption at different flow rates: (**a**) 0.25 mL·min^−1^; (**b**) 0.5 mL·min^−1^; (**c**) initial section at a rate of 0.25 mL·min^−1^. Column height—3.0 cm, *C*_0_ = 16 mmol/L; *1*—CP-1; *2*—MIP-1-2; *3*—MIP-1-4; *4*—MIP-1-6.

The mass transfer of the target substance under dynamic conditions is primarily due to the flow rate of the mobile phase. The study of the dynamics of cholesterol sorption at the flow rate of 0.25 mL·min-1 and the sorption layer height of 3.0 cm showed that the most regular sorption mode was realized on the column with the MIP-1-6 sorbent (Figure 11a,c).

The regularity of the sorption mode improved with an increase in the amount of cholesterol template added during synthesis (Figure 11c). At the same time, with an increase in the flow rate to 0.5 mL·min^−1^, the regularity of the sorption mode on MIPs did not depend on the amount of the added cholesterol template (Figure 10b). Simultaneously, more regular sorption modes were observed on all imprinted sorbents compared to sorption on CP.

An increase in the flow rate of the mobile phase led to a decrease in sorption capacities of all studied sorbents (Table 7).

At a flow rate of the mobile phase of 0.25 mL·min^−1^, an increase in sorption capacities was observed in series of imprinted polymers, and MIP-1-6 had the highest capacity. At the same time, at a given flow rate, a rapid breakthrough followed by a slow saturation of deeply located sorption centers was observed (Figure 11a). As a result, the degree of extraction of cholesterol (*R*) was less compared to *R* at the flow rate of 0.5 mL·min^−1^ (Table 7). At a flow rate of 0.25 mL·min^−1^ an increase in *R* was observed with an increase in the imprinting of the polymer network. At the same time, at the higher speed, *R* had almost the same values. For MIP-1-6, the *R* values practically did not depend on the flow rate of the mobile phase.

In addition, an increase in the flow rate of the mobile phase contributed to the exacerbation of the concentration front of cholesterol.

Imprinting factor (*IF*) values indicated the prevalence of nonspecific binding at the flow rate of the mobile phase, as the saturation of sorption centers located deeper inside the granule was achieved. An increase in the flow rate of the mobile phase to 0.5 mL·min^−1^ led to rapid saturation; as a result, the contribution of nonspecific binding decreased and *IF* values exceed 1.

**Figure 12:**
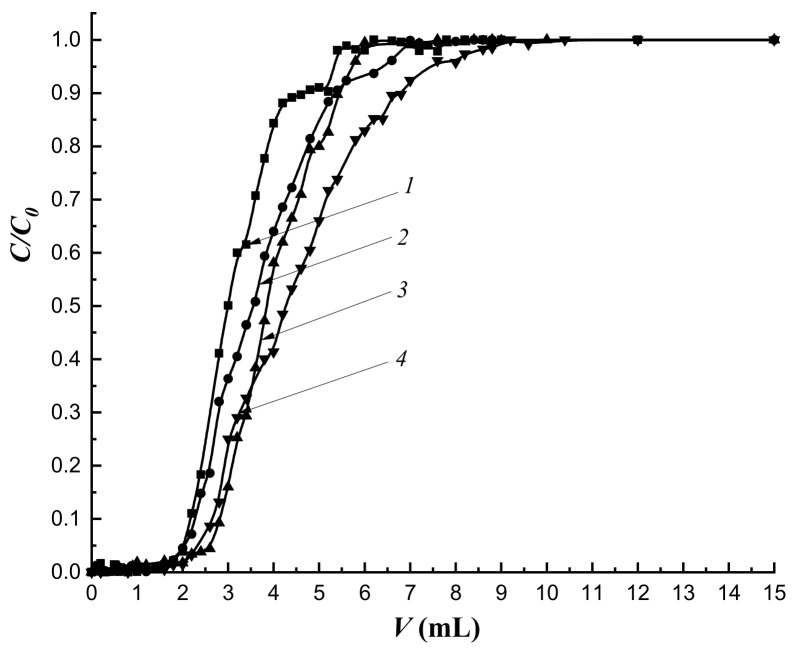
Dynamics of cholesterol sorption at the flow rate of the mobile phase 0.25 mL·min^−1^ and a column height of 4.5 cm, *C*_0_ = 16 mmol/L; *1*—CP-1; *2*—MIP-1-2; *3*—MIP-1-4; *4*—MIP-1-6.

To study the effect of the height of the sorbing layer, the flow rate was chosen as 0.25 mL·min^−1^, as values of the dynamic capacities of sorption of all studied sorbents were the highest. An increase in *H* to 4.5 cm led both to the regularization of the sorption mode on all the studied columns, and to the “blurring” of concentration fronts; this is especially noticeable on the frontal curve describing the dynamics of cholesterol sorption on MIP-1-6 (Figure 12 curve 4). However, an increase in *H* led to a decrease in sorption capacities of all organo-inorganic sorbents (Table 8). In addition, with an increase in *H*, the dependence remained: the growth of values of sorption capacities increased in series of imprinted sorbents. As a result, MIP-1-6 had the highest capacity.

The imprinting of the polymer network had practically no effect on the degree of extraction (*R*) of cholesterol at the sorption layer height of 4.5 cm.

*IF* values at this height were greater than 1 (Table 8) on all imprinted sorbents and indicated the prevalence of specific binding and an increase in the degree of imprinting in the MIP-1 series.

Thus, the regular mode of sorption dynamics, regardless of conditions, was observed during the sorption of cholesterol on columns filled with all the studied sorbents. In addition, the imprinting of the polymer network did not affect the degree of extraction of the target component from the model solution. However, the highest *q_dyn_* values were observed under conditions of the dynamic sorption process of cholesterol extraction at the flow rate of 0.25 mL·min^−1^ and H = 3.0 cm. At the same time, MIP-1-6 had the highest *IF*.

### 3.9. Study of the Selectivity of Sorption

To study the selectivity of sorption, optimal conditions for the implementation of the dynamic sorption process were chosen, namely, the flow rate of the mobile phase was 0.25 mL·min^−1^ and the height of the sorbing layer was 3.0 cm. The column filled with MIP-1-6 granules was chosen as a solid phase, as this sorbent had the highest *IF*. As noted above (Section 2.11), the object of comparison was chosen as cholic acid as the closest structural analogue of cholesterol.

**Figure 13:**
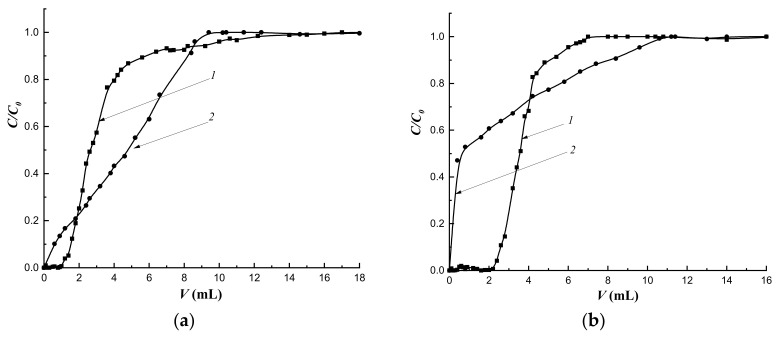
The selectivity of sorption on CP-1 (**a**) and MIP-1-6 (**b**): *1*—cholesterol, *2*—cholic acid. v = 0.25 mL·min^−1^, H = 3.0 cm.

Unlike the sorption of cholesterol, the dynamics of sorption of cholic acid was carried out in non-equilibrium modes on both CP-1 and MIP-1-6 (Figure 13). In addition, the formation of diffuse concentration fronts was observed.

The value of the parameter *α* characterizing the selectivity of the sorbent with respect to one of components for sorption on CP-1 was less than 1 (Table 9). This indicated a higher selectivity of the non-imprinted polymer to cholic acid, as cholic acid molecules were able to interact with hydroxyl groups with carbonyl groups of the polymer network. For MIP-1-6, the *α* value was greater than 1, which indicated a high selectivity with respect to the target object due to the binding of cholesterol molecules to complementary imprint sites.

Thus, the imprinting of the polymer network with 6 mol.% cholesterol led to an increase in the selectivity of the obtained polymer.

### 3.10. The Extraction of Cholesterol from Blood Plasma

**Figure 14:**
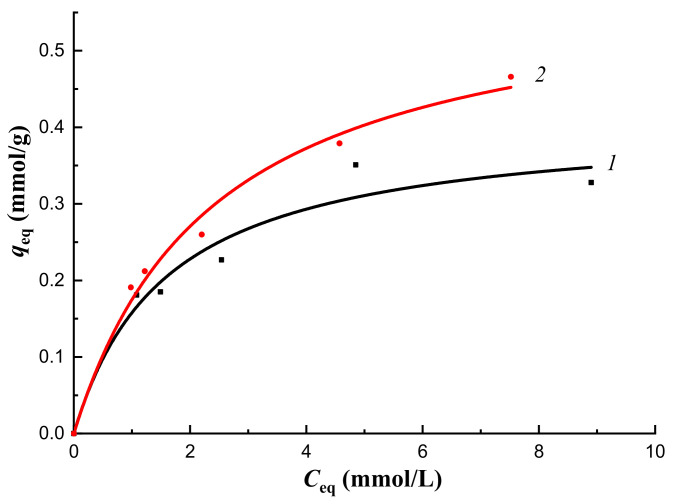
The equilibrium sorption of total cholesterol in vitro from blood plasma by sorbents CP-1 (*1*) and MIP-1-6 (*2*).

In order to prove the ability of the imprinted polymer to specifically bind a sorbent from a multicomponent mixture, the sorption of cholesterol on organo-inorganic sorbents from blood plasma was studied in vitro under conditions of the limited volume. Sorption capacities of MIP-1-6 exceeded sorption capacities of CP-1 in a wide range of concentrations (Figure 14). The degree of extraction of total cholesterol from blood plasma on CP-1 was 14%, whereas on MIP-1-6 ir was 29%.

Thus, polymer synthesized with the addition of cholesterol in the amount of 6 mol.% by Pickering emulsion polymerization was able to extract bound cholesterol from blood plasma more efficiently than CP-1. In the future, these obtained results can serve as a basis for the development of the effective dynamic process of the selective sorption of bound cholesterol from blood plasma in vivo.

## 4. Conclusions

In this study, we have identified the synthesis method of polymers, which makes it possible to obtain selective polymer sorbents based on 2-hydroxyethyl methacrylate and ethylene glycol dimethacrylate with optimal physicochemical properties—the free radical Pickering emulsion polymerization with surface imprinting.

The kinetics of swelling was shown to improve in the diffusion of n-propyl alcohol into polymer networks of sorbents synthesized by Pickering emulsion polymerization in comparison with polymer sorbents obtained by precipitation polymerization in solution. Moreover, these sorbents had polymer networks with higher porosity. It was found by scanning electron microscopy that the addition of 6 mol.% cholesterol into the polymerization mixture led to the segregation of the surface of synthesized polymer granules.

Sorbents synthesized by Pickering emulsion polymerization with the addition of cholesterol in amount of 2 mol.% and 4 mol.% had the best sorption properties in comparison with polymer sorbents synthesized by precipitation polymerization in solution. The sorption surface of polymer synthesized with the addition of cholesterol in the amount of 6 mol.% was homogeneous and had high affinity of target molecules. We showed that the imprinting of the polymer network led to the formation of more homogeneous and structurally stable networks, and an increase in the amount of template in the polymerization mixture led to an increase in the areas of available sorption surfaces in the solvated state for the target cholesterol molecule at a temperature of 37 °C, corresponding to the synthesis temperature. The study of the cholesterol dynamic sorption on polymer sorbents synthesized by Pickering emulsion polymerization allowed us to identify optimal conditions for cholesterol extraction.

Thus, Pickering emulsion polymerization led to the creation of polymers with the best physicochemical and sorption properties.

## Figures and Tables

**Table 1:** Yields of synthesized sorbents.

Sorbents	The Amount of Cholesterol mol.%	Yields, %
Method 1		
CP-1	0	67
MIP-1-2	2	76
MIP-1-4	4	82
MIP-1-6	6	83
Method 2		
CP-2	0	81
MIP-2-2	2	90
MIP-2-4	4	95
MIP-2-6	6	97

**Table 2:** Physicochemical properties of synthesized polymers.

Sorbents	*K*_sw_				*ρ*_bulk_, g/cm^3^	*ρ*_true_, g/cm^3^	*ε*, %	$W_{0,\mathrm{sum}},$ cm^3^/g
	25 °C		37 °C					
	in Water	in Propyl Alcohol	in Water	in Propyl Alcohol				
Method 1								
CP-1	2.0	2.5	2.1	3.0	0.232	0.986	76	1.5
MIP-1-2	1.9	2.0	1.9	2.5	0.165	0.952	83	2.8
MIP-1-4	2.1	2.2	2.3	2.9	0.195	0.924	81	3.1
MIP-1-6	1.8	2.2	2.0	2.6	0.170	0.926	83	3.0
Method 2								
CP-2	2.3	3.1	2.4	3.6	0.194	0.880	78	3.3
MIP-2-2	2.0	2.2	2.2	2.5	0.277	0.921	70	2.7
MIP-2-4	2.2	2.5	2.4	3.0	0.228	0.908	75	3.1
MIP-2-6	2.1	2.4	2.3	2.6	0.242	0.920	74	2.8

**Table 3:** Sorption constants calculated using the Langmuir model isotherm by the nonlinear regression method (Method 1 and Method 2).

Constants	CP	MIP-2	MIP-4	MIP-6	CP	MIP-2	MIP-4	MIP-6
	25 °C				37 °C			
Method 1								
*K_L_* (L/mmol)	-	0.55	-	0.15	2.09	-	-	0.05
*q_max_* (mmol/g)	-	0.67	-	1.78	0.27	-	-	2.20
*R* ^2^	-	0.9574	-	0.9909	0.8410	-	-	0.9956
*χ*^2^∙10^−3^	-	1.40	-	1.61	0.29	-	-	0.66
Method 2								
*K_L_* (L/mmol)	-	-	0.04	0.05	-	-	0.12	-
*q_max_* (mmol/g)	-	-	2.19	2.09	-	-	1.59	-
*R* ^2^	-	-	0.9714	0.9963	-	-	0.9740	-
*χ*^2^∙10^−3^	-	-	1.53	0.34	-	-	3.59	-

**Table 4:** Sorption constants calculated using the Freundlich model isotherm by the nonlinear regression method (Method 1 and Method 2).

Constants	CP	MIP-2	MIP-4	MIP-6	CP	MIP-2	MIP-4	MIP-6
	25 °C				37 °C			
Method 1								
*K*_F_ (L/mmol)	0.06	0.24	0.04	0.19	0.18	0.07	0.07	0.09
1*/n*_f_	1.43	0.90	0.73	1.06	0.19	1.03	1.08	1.08
*R* ^2^	0.9999	0.9997	0.7719	0.9911	0.9368	0.9953	0.9999	0.9806
*χ*^2^∙10^−5^	0.03	0.29	7.99	43.80	11.37	19.42	0.07	30.44
Method 2								
*K*_F_ (L/mmol)	0.03	0.03	0.11	0.11	-	0.33	0.12	0.07
1*/n*_f_	1.59	2.5	0.81	0.84	-	2.00	0.41	2.31
*R* ^2^	0.9996	0.9954	0.9767	0.9913	-	0.8704	0.9990	0.9993
*χ*^2^∙10^−5^	0.98	49.57	124	51.9	-	310	0.28	6.9

**Table 5:** Sorption constants calculated using the B.E.T. model isotherm by the nonlinear regression method (Method 1 and Method 2).

Constants	CP	MIP-2	MIP-4	MIP-6	CP	MIP-2	MIP-4	MIP-6
	25 °C				37 °C			
Method 1								
*K*_U_ (L/mmol)	0.04	-	-	0.03	-	-	-	0.01
*K*_L_ (L/mmol)	0.13	-	-	0.29	-	-	-	0.08
*q*_max_ (mmol/g)	0.94	-	-	0.99	-	-	-	1.36
*R* ^2^	0.9849	-	-	0.9632	-	-	-	0.9929
*χ*^2^∙10^−3^	5.35	-	-	9.00	-	-	-	0.86
Method 2								
*K*_U_ (L/mmol)	-	-	0.05	9.644 × 10^−16^	-	-	2.08 × 10^−16^	-
*K*_L_ (L/mmol)	-	-	0.32	0.05	-	-	0.12	-
*q*_max_ (mmol/g)	-	-	0.42	2.09	-	-	1.59	-
*R* ^2^	-	-	0.9931	0.9951	-	-	0.9675	-
*χ*^2^∙10^−3^	-	-	1.26	0.46	-	-	4.49	-

**Table 6:** Areas of sorption surfaces of studied polymers in a solvated state.

Sorbents	T = 25 °C			T = 37 °C		
	*q*_max_, mmol/g	*N ×* 10^20^, mol/g	*S*_SA_, m^2^/g	*q*_max_, mmol/g	*N ×* 10^20^, mol/g	*S*_SA_, m^2^/g
Method 1						
CP-1	-	-	-	0.18	1.08	61
MIP-1-2	0.55	3.31	185	0.53	3.19	179
MIP-1-4	-	-	-	0.59	3.55	199
MIP-1-6	0.99	5.96	334	-	-	-
Method 2						
CP-2	0.76	4.58	256	0.33	1.99	111
MIP-2-2	1	6.02	337	0.44	2.41	135
MIP-2-4	0.42	2.53	142	0.75	4.52	253
MIP-2-6	-	-	-	0.83	5.00	280

**Table 7:** The influence of the flow rate of the cholesterol solution on characteristics of the dynamic sorption of cholesterol, *H* = 3.0 cm.

Sorbent	*v*, mL·min^−1^	*Q*_sorb_ × 10^−2^, mmol	*q*_dyn_ × 10^−2^, mmol·mL^−1^	*IF*	*R*, %
CP-1	0.25	6.02	2.56	-	20
	0.5	4.21	1.79	-	44
MIP-1-2	0.25	5.49	2.33	0.911	23
	0.5	5.18	2.20	1.230	53
MIP-1-4	0.25	5.81	2.47	0.963	39
	0.5	5.05	2.14	1.197	57
MIP-1-6	0.25	6.55	2.78	1.086	53
	0.5	5.05	2.14	1.196	57

**Table 8:** The influence of the height of the sorption column on characteristics of the dynamic sorption of cholesterol. The flow rate of the mobile phase is 0.25 mL·min^−1^.

Sorbent	*H*, cm	*Q*_sorb_ × 10^−2^, mmol	*q*_dyn_ × 10^−2^, mmol mL^−1^	*IF*	*R*, %
CP-1	3.0	6.02	2.56	-	20
	4.5	4.95	1.40	-	52
MIP-1-2	3.0	5.52	2.35	0.916	23
	4.5	5.90	1.67	1.193	44
MIP-1-4	3.0	5.81	2.46	0.962	39
	4.5	6.32	1.79	1.278	52
MIP-1-6	3.0	6.55	2.78	1.086	53
	4.5	6.94	1.96	1.402	48

**Table 9:** Dynamic sorption characteristics of cholesterol and cholic acid on CP-1 and MIP-1-6; *v* = 0.25 mL·min^−1^, H = 3.0 cm.

Sorbent	Cholesterol		Cholic Acid		*α*
	*Q*_sorb_, × 10^−2^, mmol	*q*_chol_ × 10^−2^, mmol·mL^−1^	*Q*_sorb_, × 10^−2^, mmol	*q*_ChA_ × 10^−2^, mmol·mL^−1^	
CP-1	6.02	2.56	7.07	3.00	0.85
MIP-1-6	6.55	2.78	3.83	1.62	1.72

## Data Availability

The data presented in this study are available on request from the corresponding author.
